# Long-Term Performance of Magnetic Force Microscopy Tips Grown by Focused Electron Beam Induced Deposition

**DOI:** 10.3390/s23062879

**Published:** 2023-03-07

**Authors:** Alix Tatiana Escalante-Quiceno, Ondřej Novotný, Jan Neuman, César Magén, José María De Teresa

**Affiliations:** 1 Instituto de Nanociencia y Materiales de Aragón (INMA), CSIC-Universidad de Zaragoza, 50009 Zaragoza, Spain; 2 NenoVision s.r.o., 61200 Brno, Czech Republic; 3 Laboratorio de Microscopías Avanzadas (LMA), Universidad de Zaragoza, 50018 Zaragoza, Spain

**Keywords:** focused electron beam induced deposition, nanofabrication, magnetic tips, magnetic force microscopy

## Abstract

High-resolution micro- and nanostructures can be grown using Focused Electron Beam Induced Deposition (FEBID), a direct-write, resist-free nanolithography technology which allows additive patterning, typically with sub-100 nm lateral resolution, and down to 10 nm in optimal conditions. This technique has been used to grow magnetic tips for use in Magnetic Force Microscopy (MFM). Due to their high aspect ratio and good magnetic behavior, these FEBID magnetic tips provide several advantages over commercial magnetic tips when used for simultaneous topographical and magnetic measurements. Here, we report a study of the durability of these excellent candidates for high-resolution MFM measurements. A batch of FEBID-grown magnetic tips was subjected to a systematic analysis of MFM magnetic contrast for 30 weeks, using magnetic storage tape as a test specimen. Our results indicate that these FEBID magnetic tips operate effectively over a long period of time. The magnetic signal was well preserved, with a maximum reduction of 60% after 21 weeks of recurrent use. No significant contrast degradation was observed after 30 weeks in storage.

## 1. Introduction

Magnetic Force Microscopy (MFM) is a scanning probe technique that allows the study of magnetic samples at the nano- and micro-scale [1]. The working principle is based on the scanning of a magnetic probe, at the tip of an oscillating cantilever, over the surface of a magnetic specimen. Magnetostatic forces between the tip and the sample over the scanned area deflect the cantilever, and magnetic contrast is produced by the phase shift or resonance frequency change of the cantilever oscillation, with a typical spatial resolution of 50 nm [2,3]. MFM is a widespread magnetic characterization technique that has been used to characterize innumerable magnetic materials, such as soft magnetic samples [4,5,6], hard ferromagnets [7,8], multiferroics [9,10], and magnetic nanostructures [11,12], including novel spin textures [13,14], in ambient or vacuum conditions and even in liquid media [15,16]. To achieve high spatial resolution and sensitivity, MFM requires specialized tips that can efficiently interact with the stray magnetic fields produced by the specimen. The most common MFM tips are non-magnetic atomic force microscopy (AFM) probes coated with a layer of a ferromagnetic material, which can be cobalt- and nickel-based alloys, magnetic multilayers and iron oxides, among others [17,18,19,20]. The quality of the MFM images is strongly dependent on the physical dimensions of the tip, including its size and shape, as well as its magnetic properties such as saturation magnetization, remanence, and coercivity.

The magnetic sensitivity of MFM improves with the increasing thickness of the ferromagnetic layer thanks to a greater interacting magnetic volume, while the lateral resolution decreases due to the enlarged diameter and size of the tip [21]. Different solutions have been reported to simultaneously improve resolution and sensitivity. For instance, the deposition of non-magnetic material on the apex of the probe by electron beam lithography, subsequently coating one side of the tip with a ferromagnetic film by evaporation [22,23]; FIB milling to reduce the diameter of a previously coated AFM tip [24,25]; the growth of carbon nanotubes coated with ferromagnetic films [26,27], or the sputtering deposition only on one side of the tip pyramid [28]. These approaches have yielded good results in terms of resolution and sensitivity. However, their production is complex and time-consuming, and involves laborious multi-step procedures.

**Figure 1:**
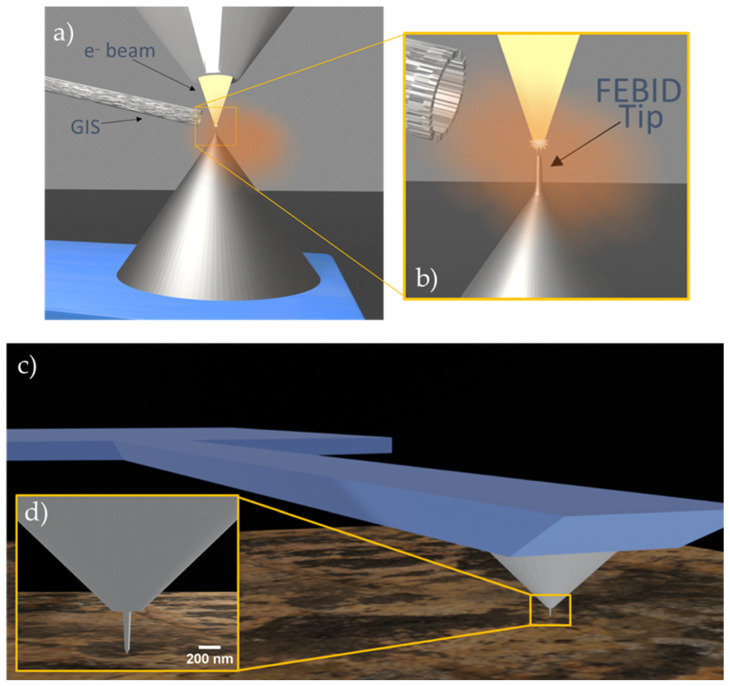
Schematic representation of the FEBID magnetic tip during the growth process (**a**,**b**) and the MFM measurement (**c**,**d**). (**a**) Overview of the deposition process of Fe tips by FEBID on the apex of a commercial AFM tip. (**b**) Detailed view of the growth process of the Fe tip, in which the GIS delivers the gas precursor during the electron beam irradiation, producing a vertical deposit on the apex of the AFM tip. (**c**) Wide view of the functionalized cantilever. (**d**) Detail of the cantilever tip, which shows the size ratio of AFM tip and the FEBID deposit. Commercial tips have an apex diameter of approximately 100 nm, whereas the tip of FEBID-grown Fe nanowire is around 15 nm wide.

An alternative nanofabrication method has been developed to functionalize scanning probes for high-sensitivity and high-resolution MFM measurements by Focused Electron Beam Induced Deposition (FEBID) [29,30,31]. FEBID is a nanofabrication technique that uses a focused electron beam to selectively deposit material onto a surface with nanometer precision. This technique is based on the principle of electron-induced dissociation of precursor molecules. A gas injector system (GIS) supplies a flux of gas precursor over a substrate placed in a vacuum chamber, and the adsorbed molecules are decomposed by the irradiation of a focused electron beam, producing the local deposition of a solid material as shown in Figure 1a,b. The deposited material can be patterned by controlling the electron beam trajectory, allowing for the fabrication of complex 2D and 3D structures at the nanoscale. FEBID is a versatile technique that can deposit a wide range of functional materials, including metals, insulators, and semiconductors, making it suitable for a wide range of applications [32]. In particular, ferromagnetic deposits can be synthesized by FEBID using different gaseous organometallic precursors containing magnetic elements such as Co, Fe, or Ni [33,34,35,36,37]. Numerous studies have shown that it is possible to produce high-purity 2D and 3D magnetic nanostructures using FEBID [37]. Their excellent magnetic properties and tunable architecture make them great candidates for a variety of uses in different technological fields, such as data storage and logics, sensing, spintronic, and biological applications [38,39,40,41]. 

One application that takes full advantage of the capabilities of FEBID is the functionalization of MFM probes with 3D ferromagnetic deposits, especially nanowires. The use of FEBID allows for precise control over the size, shape, and magnetic properties of a magnetic deposit grown at the tip of an MFM cantilever, which can be optimized for specific applications. For example, it is possible to grow very sharp and narrow (<50 nm) nanowires with a high aspect ratio to maximize the coercive field, or with tailored magnetization to tune the magnetic sensitivity. The magnetic properties of 3D-FEBID magnetic tips for MFM applications have been thoroughly investigated by Jaafar et al. and Pablo-Navarro et al. [41,42]. The switching field has been studied, and it has been found that the MFM magnetic contrast is reversed above 900 Oe for Fe tips and between 550–600 for Co tips. This indicates a high coercive field compared to commercial magnetic tips, which is around 200–400 Oe [41]. The magnetic field of the nanowire tip has also been analyzed using electron holography to determine the tip–sample interaction. It has been shown that it is possible to control the stray field by modifying the shape and composition of the tip. At the apex the magnetic field depends strongly on the tip diameter, from 80 mT to 260 mT for tip diameters ranging 30–70 nm, while at a distance of 50 nm the stray field presents quite similar values, from 50 to 80 mT. It should be noted that a detailed knowledge of the stray field of the tip is key to obtaining quantitative measurements of the magnetization of the sample [41]. It has been found that tips with high aspect-ratio Fe nanowires interfere weakly with the magnetic structure of the sample, even in soft magnetic structures [42,43]. Because of the sharp end of the FEBID magnetic tips, it is possible to obtain images with a high lateral resolution (down to 20 nm) compared to commercial tips. Furthermore, the tips have been tested in aqueous media and demonstrated performance similar to air conditions, which is of great interest for the study of magnetic biological samples [42,43]. The relative dimensions of the AFM cantilevers used and the FEBID-grown magnetic tips are illustrated in Figure 1c,d.

Due to the minute dimensions, high aspect ratio, and increased manufacturing cost with respect to the commercial probes, the robustness and operational lifetime of the 3D-FEBID magnetic tips are a matter of concern. Furthermore, both commercial and FEBID-grown MFM probes are susceptible to damage during human manipulation, which is an additional consideration for tip performance. Here we present a study designed to test the durability of these magnetic tips grown by FEBID by analyzing the evolution of the magnetic contrast provided upon regular use for MFM experiments. For this purpose, we will use Akiyama probes marketed by NenoVision s.r.o. Akiyama probes are self-detecting and self-exciting probes for dynamic AFM, requiring neither optical detection nor an external excitation, and occupying a small volume over the sample, which makes them very attractive for different applications [44].

## 2. Materials and Methods

### 2.1. FEBID Fe-Based Magnetic Tips Fabrication

Fe deposits were grown with a commercial Thermo Fisher Helios Nanolab 600 dual beam system, installed in the Laboratorio de Microscopías Avanzadas (LMA) at INMA (Zaragoza, Spain), using an organometallic gas precursor of Fe_2_(CO)_9_. An electron beam voltage of 30 kV was used to obtain sharp tips. The electron beam current was kept constant at 0.69 nA to obtain tip diameters of ~75 nm. The base pressure inside the working chamber was 7 × 10^−7^ mbar. During gas injection, the chamber pressure increased with respect to the base pressure, which is directly related to the flow of precursor into the chamber. Thus, the gas flux was monitored by the precursor pressure (ΔP), calculated as the difference between the growth pressure and the base pressure. The precursor pressure decreases with time, so the irradiation time was varied between 18 and 31 s during the deposition runs in order to keep the deposit dimensions constant, obtaining tips with heights of around 650 nm.

The FEBID-Fe tips were deposited on non-magnetic commercial Akiyama probes. These AFM sensors have a quartz tuning fork system attached to a silicon cantilever with the following specifications: length = 310 μm, width = 30 μm, spring constant = 5 Nm^−1^, resonance frequency = 45 kHz. A careful alignment of the dual beam system was necessary to produce the deposit accurately on the apex of the commercial Akiyama tips. Ten tips were fabricated with an aspect ratio greater than 7, with the exception of probes 1 and 2 which had an aspect ratio of approximately 4.5.

### 2.2. MFM Measurements

The MFM measurements were carried out at the Nenovision s.r.o. facility in Brno, Czech Republic. A LiteScopeTM microscope, designed to be easily integrated into a scanning electron microscope (SEM), was used. Three test groups were established with three probes each. Each group was measured approximately every 6 weeks. An additional probe was used as a control probe: it was tested on week 1, then stored in its gel-pack box inside the original antistatic bag, and finally measured again on week 30. The probes were not magnetized before the measurements.

Dual-pass MFM was carried out in a low vacuum (~0.13 mbar) environment. Scanning parameters were defined as follows: scan size = 10 × 10 µm, image size = 512 × 512 pixels, speed = 20 μm/s, second pass speed = 10 μm/s, frequency set point = 10 Hz, amplitude set point first pass = 0.4 V, excitation amplitude second pass = 0.1 V, lift height during second pass = 100 nm. The reference sample used for the tests was a magnetic storage tape provided by Bruker (model: MFMSAMPLE), which presents magnetic stripes of a few microns width over the scan area. In dual-pass MFM, each line is scanned twice. The first pass was performed in tapping mode to map the topography of the sample. For the second pass in non-contact mode, the tip was moved to a specified distance away from the sample surface (lift height), where the van der Waals forces are negligible compared to the magnetic forces. Magnetic tip–sample interaction was monitored during the second pass by the phase signal, i.e., the phase shift of the cantilever oscillation caused by the magnetic interaction. Topography images were processed using row alignment (median of difference) and subtracting the quadratic background. For the MFM images we used row alignment (median of difference) and subtracted a plane background.

## 3. Results

### 3.1. FEBID-Tips Fabrication

**Figure 2:**
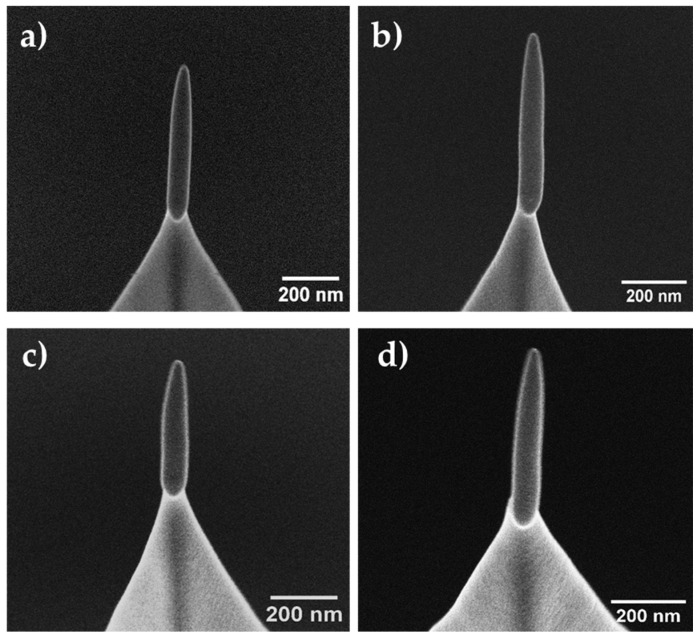
SEM images of FEBID Fe-based magnetic probes grown on the apex of commercial Akiyama probes: (**a**) tip 5, (**b**) tip 7, (**c**) tip 8, and (**d**) tip 10. The length and diameter of the tips were around 650 nm and 75 nm, respectively.

SEM images of some FEBID magnetic tips grown on Akiyama AFM probes using the gas precursor Fe_2_(CO)_9_ are depicted in Figure 2. FEBID growth enabled us to obtain tips of different heights and diameters with high accuracy and reproducibility. By varying the beam voltage, it is possible to modify the shape of the tip, producing sharp or blunt tips. Sharper tips grown at 30 kV have shown better performance during MFM measurements than blunt tips [43]. Table 1 summarized the basic growth parameters (precursor pressure and deposition time) and geometrical dimensions of the batch of functionalized tips used for the durability test. The dimensions of tips 3–10 were similar, with diameters of ~75 nm and lengths of ~650 nm, and variation coefficients in diameter and length of 2% and 5%, respectively. Tips 1 and 2 were significantly different, shorter and narrower than the rest of the tips, as they were used to find the optimal growth conditions. The growth of the Fe-based tips takes about 30 s of electron beam irradiation. Producing ten tips typically takes about two hours of work. The Fe content of the 3D-FEBID magnetic tips is ~70% at., with the remaining ~30% at. corresponding to carbon and oxygen in similar proportions, derived from the residues of the precursor gas [43].

### 3.2. MFM Results

**Figure 3:**
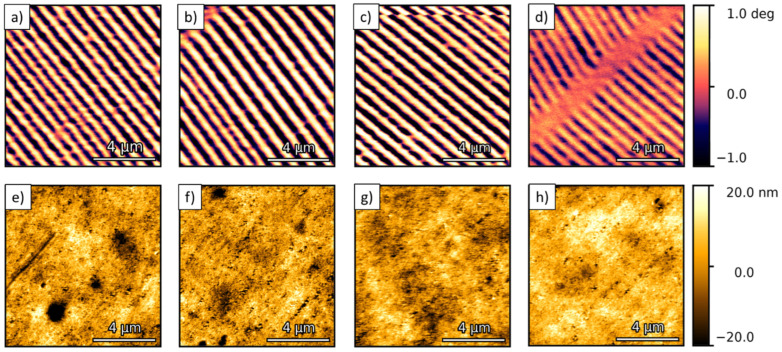
MFM (**a**–**d**) and topographic AFM (**e**–**h**) images measured with magnetic tip 4 in week 1 (**a**,**e**), week 7 (**b**,**f**), week 13 (**c**,**g**) and week 21 (**d**,**h**). The MFM images taken with tip 4 show a decrease in magnetic contrast in week 21 compared to the images obtained in the previous weeks.

To analyze the performance of the magnetic tips during their lifetime, MFM measurements were carried out using a magnetic storage tape “MFMSAMPLE” as a reference sample. Measurements were carried out in four test groups: N1, PG1, PG2, and PG3. The data from these samples are included in Table 2. The study of the N1 and PG1 groups started at the NenoVision facilities two weeks after they were synthesized at INMA, while the measurements of the PG2 and PG3 groups started four and six weeks after their growth, respectively. The measurements were performed in dual-pass MFM. The measurement conditions were kept constant for each probe. Figure 3 depicts the magnetic (MFM) and topographic (AFM) images taken with tip 4 as a function of time. Well-defined magnetic domains of 0.380 ± 0.001 μm with a contrast amplitude of 1 deg are observed in the MFM images. From the AFM images, a sample roughness of ~5 nm is observed. A qualitative analysis of the images shows a degradation of the magnetic signal of tip 4 from 2.2 ± 0.3 deg at week 1 to 1.3 ± 0.3 deg at week 21. However, the magnetic domains are still well resolved.

**Figure 4:**
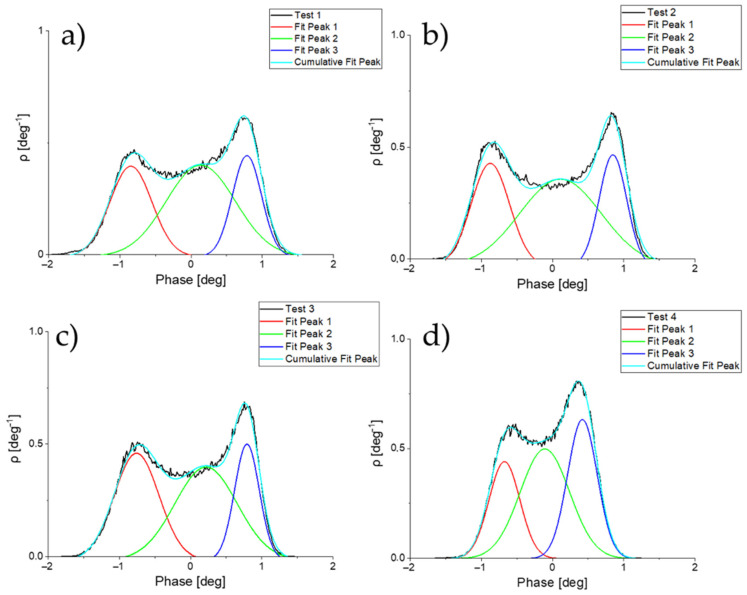
Representation of the computational extraction of the height distribution function which represents the maximum and minimum MFM contrast obtained from the magnetic tip 10 at week 1 (**a**), 7 (**b**), 13 (**c**), and 21 (**d**). The blue and red curves represent the maximum and minimum phase values.

The evolution of the magnetic contrast of the tips with time and use was analyzed following a semi-quantitative method based on the computational extraction from the MFM image of the height distribution, which is the density function of the normalized histograms of the heights, as shown in Figure 4. The data were then plotted and fitted to three Gaussian curves to obtain the magnetic contrast and FWHM values. The Gaussian curves correspond to the positive magnetic contrast of the first magnetic domain (red), the contribution of the magnetic domain walls (green), and the negative magnetic contrast of the second magnetic domain (blue). The apex of the positive and negative curves is the average value of the maximum and minimum contrast. This means that each peak in the graph represents the maximum or minimum magnetic phase detected by each tip when scanning the sample. The difference between the negative and positive contrast of each MFM measurement is related to the phase amplitude of the cantilever oscillation, which is directly proportional to the tip–sample interaction. The reduction in the magnetic signal over time will be reflected in the reduction in the phase amplitude with respect to the first measurement. The Gwyddion v2.61 software package was used to extract the density function from the MFM images. 

Table 2 shows a compilation of the change in magnetic contrast of each tip over time. The data represented in the table are the average value of the differences between the maximum and minimum values of the magnetic phase amplitudes. Tips 5, 8, and 9 were mechanically damaged during handling at different stages of the durability test. Tips 2, 3, and 7 lost the magnetic signal after the second measurement. However, no drastic drop in the magnetic signal was witnessed in the last valid measurement, suggesting that the magnetic signal had not degraded by that time. Tips 4, 6, and 10 passed all tests with good performance.

**Figure 5:**
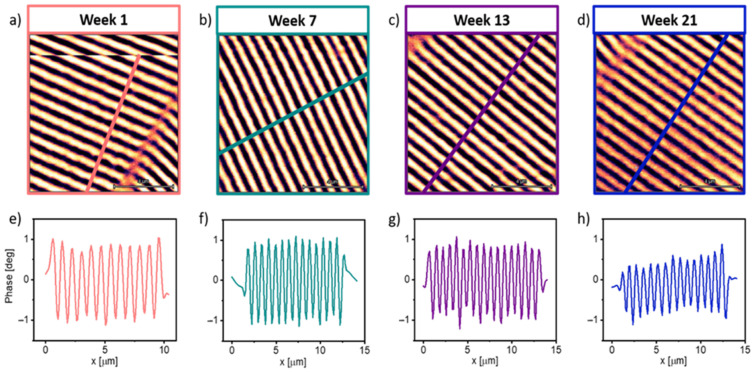
MFM images (**a**–**d**) of the magnetic storage tape reference sample obtained by tip 10 and their corresponding line profiles of magnetic contrast (**e**–**h**). These measurements were carried out over a period of 21 weeks. After 21 weeks it is observed that the magnetic signal is still high. In the profiles of week 1 (**a**), week 7 (**b**) and week 13 (**c**) it is observed that the magnetic domain contrasts have an amplitude of ~1°, and in the profile of week 21 a lower contrast is observed with greater variation.

A reduction of 30% in phase amplitude was found after 20 weeks of use for tips 6 and 10, while tip 4 showed a reduction of 60% at week 21. This result suggests that the magnetic signal of the tips is conserved well over time and use, with no significant decay in the magnetic signal and remaining in the same order of magnitude. Figure 5 shows the profiles of the MFM images taken with tip 10. The profiles were taken at 90 degrees with respect to the orientation of the magnetic domains. It is observed that up to week 13 the phase amplitude was maintained at ±1 deg; the reduction in the phase is clearly seen at week 21, which is consistent with the results obtained from the histograms shown in Table 2.

We also found that tip 1, used as a control probe, did not show a significant reduction in magnetic contrast 30 weeks after the initial test, with only a 7% reduction in magnetic contrast, thus evidencing that the performance of the FEBID-functionalized probe remains very high upon storage.

**Figure 6:**
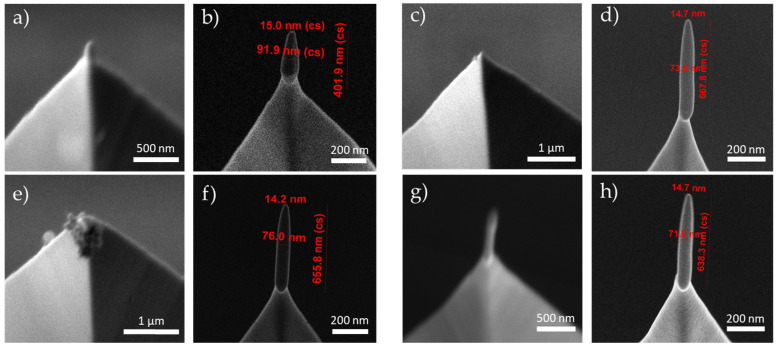
SEM images of FEBID Fe-based magnetic probes after (**a**,**c**,**e**,**g**) and before (**b**,**d**,**f**,**h**) the durability tests: (**a**,**b**) tip 1, (**c**,**d**) tip 5, (**e**,**f**) tip 7, and (**g**,**h**) tip 10. After 21 weeks it is observed that tip 1 and tip 10 are still on the top of the Akiyama probe, while tip 5 and tip 7 are no longer in the upper part of the Akiyama probe.

SEM images (Figure 6) were obtained to verify the tips after all the MFM measurements were concluded. Tips 1 and 10 remained attached to the apex of the cantilever, with no apparent geometrical modification or degradation. It is worth noting that while tip 10 showed a significant reduction in magnetic signal, control tip 1 showed no magnetic signal decay during storage. Tips 5 and 7 were not found, which is consistent with the results shown in Table 2. Tip 5 was reported to show evident mechanical damage during operation. In the case of tip 7 no mishandling was observed, even though it suffered a complete loss of magnetic signal at week 14. Thus, from the post-measurement images it can be concluded that this loss of magnetic signal was also due to the breaking of the FEBID tip, and not to the progressive deterioration of its magnetization.

## 4. Conclusions and Outlook

Previous works have shown that scanning probes functionalized with 3D-FEBID magnetic tips can be used for simultaneous topographic and magnetic measurements with low non-magnetic tip–sample interaction, high lateral resolution, and a high coercive field of the tips. Here we tested the long-term performance and robustness of FEBID Fe-based magnetic tips by measuring the same reference sample for several weeks with the same probes. We semi-quantitatively measured the change in magnetic contrast over time, based on the difference between the average values of the maximum and minimum amplitudes of the magnetic phase. Our results suggest that the 3D-FEBID Fe-based magnetic tips exhibit robust performance for a long lifetime of up to 30 weeks. The MFM measurements performed with these tips conserved the magnetic signal well over time and use, with a maximum reduction of 60% in magnetic signal with use after more than 20 weeks and no significant degradation of magnetic signal during storage for 30 weeks.

These results highlight the potential of FEBID-grown magnetic tips for reliable, high-resolution MFM probes for laboratory-scale and industrial use. The versatility of the technique allows the growth of tips of different dimensions on different types of probes with optical detection or self-sensing cantilevers. Due to its mechanical stability, resolution, sensitivity, and performance over the long term, it is possible to perform MFM measurements in air, vacuum, and even liquid media, opening up new possibilities for the study of magnetic bioparticles. Furthermore, thanks to the low magnetic tip–sample interaction, it is possible to analyze both magnetically soft and magnetically hard samples.

## Figures and Tables

**Table 1:** Main growth parameters and geometrical dimensions of the Fe tips grown by FEBID. Deposition time as a function of precursor pressure (ΔP) is shown, as well as diameter, lengths and aspect ratio obtained under each condition. Precursor pressure is calculated as the difference between pressure during growth and base pressure.

Tip	ΔP (10^−6^ mbar)	Deposition Time (s)	Length (nm)	Diameter (nm)	Aspect Ratio
1	5.4	18	401.9	91.9	4.4
2	4.1	20	354.7	76.5	4.6
3	4.6	28	626.6	75.0	8.4
4	4.3	28	670.5	71.7	9.4
5	3.4	29	655.8	76.0	8.6
6	4.0	29	626.1	75.4	8.3
7	3.4	29	667.8	73.8	9.0
8	2.9	29	550.0	75.0	7.3
9	2.7	31	634.5	72.8	8.7
10	2.3	31	638.3	71.6	8.9

**Table 2:** Evolution of the magnetic contrast (in deg, as explained in the text) over time for the tested FEBID magnetic tips. Tip 1 was studied the first week, stored for 30 weeks, and then measured again at the end of the test. The red circles represent tests in which no results were obtained due to mechanical damage of the tip or loss of the magnetic signal, as noted in the Comments column. It is worth mentioning that the tips were not externally magnetized at any time.

			Weeks					
		Tip	1	7	13	21	30	Comments
Phase Shift [deg]	N1	1	0.41 ± 0.02				0.38 ± 0.01	The tip passed all the tests
	PG1	2	0.28 ± 0.04	0.19 ± 0.16	o	o		MFM signal lost
		3	1.53 ± 0.02	1.48 ± 0.02	o	o		MFM signal lost
		4	1.17 ± 0.01	1.52 ± 0.02	1.49 ± 0.01	0.48 ± 0.01		The tip passed all the tests
	PG2	5	1.86 ± 0.03	1.31 ± 0.01	o	o		Tip mechanically damaged
		6	1.81 ± 0.01	1.45 ± 0.01	1.29 ± 0.01	1.27 ± 0.03		The tip passed all the tests
		7	2.44 ± 0.03	1.39 ± 0.02	o	o		MFM signal lost
	PG3	8	o	o	o	o		Tip mechanically damaged
		9	1.48 ± 0.01	1.46 ± 0.01	1.31 ± 0.01	o		Tip mechanically damaged
		10	1.63 ± 0.01	1.72 ± 0.01	1.55 ± 0.01	1.09 ± 0.02		The tip passed all the tests

## Data Availability

Data is available under reasonable request.

## References

[1] Kazakova O., Puttock R., Barton C., Corte-León H., Jaafar M., Neu V., Asenjo A. (2019). Frontiers of magnetic force microscopy. J. Appl. Phys..

[2] Schwarz A., Wiesendanger R. (2008). Magnetic sensitive force microscopy. Nano Today.

[3] Feng Y., Vaghefi P.M., Vranjkovic S., Penedo M., Kappenberger P., Schwenk J., Zhao X., Mandru A.-O., Hug H. (2022). Magnetic force microscopy contrast formation and field sensitivity. J. Magn. Magn. Mater..

[4] Yagil A., Almoalem A., Soumyanarayanan A., Tan A.K.C., Raju M., Panagopoulos C., Auslaender O.M. (2018). Stray field signatures of Néel textured skyrmions in Ir/Fe/Co/Pt multilayer films. Appl. Phys. Lett..

[5] Rawlings C., Durkan C. (2012). Performing quantitative MFM measurements on soft magnetic nanostructures. Nanotechnology.

[6] Wood R., Smith N. (2021). Fields from a Magnetized Conical Shell and Quantitative Magnetic Force Microscopy. IEEE Trans. Magn..

[7] Vergara J., Favieres C., Madurga V. (2022). Magnetic domain configurations of pulsed laser deposited MnBi hard magnetic films. J. Magn. Magn. Mater..

[8] Salaheldeen M., Vega V., Ibabe A., Jaafar M., Asenjo A., Fernandez A., Prida V.M. (2018). Tailoring of Perpendicular Magnetic Anisotropy in Dy13Fe87 Thin Films with Hexagonal Antidot Lattice Nanostructure. Nanomaterials.

[9] Ghidini M., Maccherozzi F., Dhesi S.S., Mathur N.D. (2022). XPEEM and MFM Imaging of Ferroic Materials. Adv. Electron. Mater..

[10] Heczko O. (2020). Antiphase boundaries in Ni-Mn-Ga ordered compound. AIP Adv..

[11] Ehrmann A., Blachowicz T. (2021). Magnetic Force Microscopy on Nanofibers—Limits and Possible Approaches for Randomly Oriented Nanofiber Mats. Magnetochemistry.

[12] Angeloni L., Passeri D., Corsetti S., Peddis D., Mantovani D., Rossi M. (2017). Single nanoparticles magnetization curves by controlled tip magnetization magnetic force microscopy. Nanoscale.

[13] Stepanova M., Masell J., Lysne E., Schoenherr P., Köhler L., Paulsen M., Qaiumzadeh A., Kanazawa N., Rosch A., Tokura Y. (2022). Detection of Topological Spin Textures via Nonlinear Magnetic Responses. Nano Lett..

[14] Arekapudi S.S.P.K., Böhm B., Ramasubramanian L., Ganss F., Heinig P., Stienen S., Fowley C., Lenz K., Deac A.M., Albrecht M. (2021). Direct imaging of distorted vortex structures and magnetic vortex annihilation processes in ferromagnetic/antiferromagnetic disk structures. Phys. Rev. B.

[15] Schwenk J., Marioni M., Romer S., Joshi N.R., Hug H.J. (2014). Non-contact bimodal magnetic force microscopy. Appl. Phys. Lett..

[16] Vokoun D., Samal S., Stachiv I. (2022). Magnetic Force Microscopy in Physics and Biomedical Applications. Magnetochemistry.

[17] Suzuki R., Ishihara S., Ohtake M., Futamoto M. (2014). Fabrication of Tips for Magnetic Force Microscopy Employing Magnetic Multilayer Structures. Key Eng. Mater..

[18] Ishihara S., Ohtake M., Futamoto M. (2014). Switching fields of high-resolution magnetic force microscope tips coated with Co, Co75Pt10Cr15, Co75Pt25, and Co50Pt50films. EPJ Web Conf..

[19] Koblischka M., Kirsch M., Wei J., Sulzbach T., Hartmann U. (2007). Preparation of ferrite-coated MFM cantilevers. J. Magn. Magn. Mater..

[20] Sungthong A., Ruksasakchai P., Saengkaew K., Cheowanish I., Damrongsak B. (2017). Response of Magnetic Force Microscopy Probes under AC Magnetic Field. J. Phys. Conf. Ser..

[21] Akdogan O., Akdogan N.G. (2021). SmCo-based MFM probes with high switching fields. J. Magn. Magn. Mater..

[22] Fischer P.B. (1993). Ultrahigh resolution magnetic force microscope tip fabricated using electron beam lithography. J. Vac. Sci. Technol. B Microelectron. Nanometer Struct..

[23] Koblischka M., Hartmann U., Sulzbach T. (2003). Improvements of the lateral resolution of the MFM technique. Thin Solid Film..

[24] Phillips G.N., Siekman M., Abelmann L., Lodder J.C. (2002). High resolution magnetic force microscopy using focused ion beam modified tips. Appl. Phys. Lett..

[25] Gao L., Yue L., Yokota T., Skomski R., Liou S., Takahoshi H., Saito H., Ishio S. (2004). Focused Ion Beam Milled CoPt Magnetic Force Microscopy Tips for High Resolution Domain Images. IEEE Trans. Magn..

[26] Kuramochi H., Manago T., Koltsov D., Takenaka M., Iitake M., Akinaga H. (2007). Advantages of CNT–MFM probes in observation of domain walls of soft magnetic materials. Surf. Sci..

[27] Yoshida N., Arie T., Akita S., Nakayama Y. (2002). Improvement of MFM Tips Using Fe-Alloy-Capped Carbon Nanotubes. Phys. B Condens. Matter.

[28] Iglesias-Freire O., Jaafar M., Berganza E., Asenjo A. (2016). Customized MFM probes with high lateral resolution. Beilstein J. Nanotechnol..

[29] Utke I., Hoffmann P., Berger R., Scandella L. (2002). High-resolution magnetic Co supertips grown by a focused electron beam. Appl. Phys. Lett..

[30] Gavagnin M., Wanzenboeck H.D., Belic D., Shawrav M.M., Persson A., Gunnarsson K., Svedlindh P., Bertagnolli E. (2013). Magnetic force microscopy study of shape engineered FEBID iron nanostructures. Phys. Status Solidi A.

[31] Gavagnin M., Wanzenboeck H.D., Wachter S., Shawrav M.M., Persson A., Gunnarsson K., Svedlindh P., Stöger-Pollach M., Bertagnolli E. (2014). Free-Standing Magnetic Nanopillars for 3D Nanomagnet Logic. ACS Appl. Mater. Interfaces.

[32] Huth M., Porrati F., Dobrovolskiy O. (2018). Focused electron beam induced deposition meets materials science. Microelectron. Eng..

[33] Idigoras O., Nikulina E., Porro J.M., Vavassori P., Chuvilin A., Berger A. (2014). FEBID fabrication and magnetic characterization of individual nano-scale and micro-scale Co structures. Nanofabrication.

[34] Pérez-Roldán M.J., Tatti F., Vavassori P., Berger A., Chuvilin A. (2015). Segregation of materials in double precursor electron-beam-induced-deposition: A route to functional magnetic nanostructures. Nanotechnology.

[35] Córdoba R., Barcones B., Roelfsema E., Verheijen M.A., Mulders J.J.L., Trompenaars P.H.F., Koopmans B. (2016). Functional nickel-based deposits synthesized by focused beam induced processing. Nanotechnology.

[36] Porrati F., Pohlit M., Müller J., Barth S., Biegger F., Gspan C., Plank H., Huth M. (2015). Direct writing of CoFe alloy nanostructures by focused electron beam induced deposition from a heteronuclear precursor. Nanotechnology.

[37] Magén C., Pablo-Navarro J., De Teresa J. (2021). Focused-Electron-Beam Engineering of 3D Magnetic Nanowires. Nanomaterials.

[38] Preischl C., Le L.H., Bilgilisoy E., Vollnhals F., Gölzhäuser A., Marbach H. (2020). Controlled Electron-Induced Fabrication of Metallic Nanostructures on 1 nm Thick Membranes. Small.

[39] Skoric L., Donnelly C., Hierro-Rodriguez A., Sandoval M.A.C., Ruiz-Gómez S., Foerster M., Niño M.A., Belkhou R., Abert C., Suess D. (2022). Domain Wall Automotion in Three-Dimensional Magnetic Helical Interconnectors. ACS Nano.

[40] Mattiat H., Rossi N., Gross B., Pablo-Navarro J., Magén C., Badea R., Berezovsky J., De Teresa J.M., Poggio M. (2020). Nanowire Magnetic Force Sensors Fabricated by Focused-Electron-Beam-Induced Deposition. Phys. Rev. Appl..

[41] Jaafar M., Pablo-Navarro J., Berganza E., Ares P., Magén C., Masseboeuf A., Gatel C., Snoeck E., Gómez-Herrero J., de Teresa J.M. (2020). Customized MFM probes based on magnetic nanorods. Nanoscale.

[42] Pablo-Navarro J., Sangiao S., Magén C., de Teresa J.M. (2021). Magnetic Functionalization of Scanning Probes by Focused Electron Beam Induced Deposition Technology. Magnetochemistry.

[43] Berganza E., Jaafar M., Fernandez-Roldan J.A., Goiriena-Goikoetxea M., Pablo-Navarro J., García-Arribas A., Guslienko K., Magén C., De Teresa J.M., Chubykalo-Fesenko O. (2020). Half-hedgehog spin textures in sub-100 nm soft magnetic nanodots. Nanoscale.

[44] Stiller M., Barzola-Quiquia J., Esquinazi P.D., Sangiao S., De Teresa J.M., Meijer J., Abel B. (2017). Functionalized Akiyama tips for magnetic force microscopy measurements. Meas. Sci. Technol..

